# Precision therapeutic targets for COVID-19

**DOI:** 10.1186/s12985-021-01526-y

**Published:** 2021-03-29

**Authors:** Zachary A. Krumm, Grace M. Lloyd, Connor P. Francis, Lith H. Nasif, Duane A. Mitchell, Todd E. Golde, Benoit I. Giasson, Yuxing Xia

**Affiliations:** 1 Department of Neuroscience, College of Medicine, University of Florida, 1275 Center Drive, Gainesville, FL 32610 USA; 2 Center for Translational Research in Neurodegenerative Disease, College of Medicine, University of Florida, Gainesville, FL 32610 USA; 3 College of Medicine, McKnight Brain Institute, University of Florida, Gainesville, FL 32610 USA; 4 Lillian S. Wells Department of Neurosurgery, University of Florida, Gainesville, FL 32610 USA; 5 UF Clinical and Translational Science Institute, University of Florida, Gainesville, FL 32610 USA

**Keywords:** SARS-CoV-2, COVID-19, M^Pro^, Main protease, RNA-dependent RNA polymerase, Spike protein, Therapy

## Abstract

Beginning in late 2019, severe acute respiratory syndrome coronavirus 2 (SARS-CoV-2) emerged as a novel pathogen that causes coronavirus disease 2019 (COVID-19). SARS-CoV-2 has infected more than 111 million people worldwide and caused over 2.47 million deaths. Individuals infected with SARS-CoV-2 show symptoms of fever, cough, dyspnea, and fatigue with severe cases that can develop into pneumonia, myocarditis, acute respiratory distress syndrome, hypercoagulability, and even multi-organ failure. Current clinical management consists largely of supportive care as commonly administered treatments, including convalescent plasma, remdesivir, and high-dose glucocorticoids. These have demonstrated modest benefits in a small subset of hospitalized patients, with only dexamethasone showing demonstrable efficacy in reducing mortality and length of hospitalization. At this time, no SARS-CoV-2-specific antiviral drugs are available, although several vaccines have been approved for use in recent months. In this review, we will evaluate the efficacy of preclinical and clinical drugs that precisely target three different, essential steps of the SARS-CoV-2 replication cycle: the spike protein during entry, main protease (M^Pro^) during proteolytic activation, and RNA-dependent RNA polymerase (RdRp) during transcription. We will assess the advantages and limitations of drugs that precisely target evolutionarily well-conserved domains, which are less likely to mutate, and therefore less likely to escape the effects of these drugs. We propose that a multi-drug cocktail targeting precise proteins, critical to the viral replication cycle, such as spike protein, M^Pro^, and RdRp, will be the most effective strategy of inhibiting SARS-CoV-2 replication and limiting its spread in the general population.

**Supplementary Information:**

The online version contains supplementary material available at 10.1186/s12985-021-01526-y.

## Introduction

The novel betacoronavirus, commonly referred to as coronavirus disease 2019 (COVID-19) and severe acute respiratory syndrome coronavirus 2 (SARS-CoV-2), emerged in the city of Wuhan within the Hubei Province of China in late 2019 [1–3]. As of February 22, 2021, an estimated 111 million individuals have been infected with SARS-CoV-2 globally, with over 2.47 million deaths attributed to infection worldwide. At least 110 countries have reported > 10,000 confirmed cases, with the current number of worldwide cases increasing at a rate of over 400,000–500,000 cases per day [4]. SARS-CoV-2 shares significant homology with multiple betacoronaviruses that have produced outbreaks of viral pneumonias, the most notable being severe acute respiratory syndrome (SARS) in 2003 and Middle Eastern Respiratory Syndrome (MERS) beginning in 2012 [5–7]. Viruses in the SARS-CoV and MERS-CoV families share significant homology with betacoronaviruses that commonly circulate among bat populations, and each appears to have garnered infectivity for humans following transmission through an intermediate host—civets (*Paguma larvata*) in SARS-CoV, pangolins (*Pholidota*) in SARS-CoV-2 [7–9] and camels in MERS-CoV [8–11]. Symptoms of these betacoronaviruses include fever, cough, dyspnea, fatigue, muscle weakness, headache, nausea, and diarrhea [12, 13]. Loss of smell has been reported in patients with SARS-CoV-2 [12], and, like other betacoronaviruses, severe cases progress to a pneumonia, myocarditis, cytokine storm, hypercoagulability, acute respiratory distress syndrome (ARDS), septic shock, complete respiratory failure, multiple organ failure, and a high rate of fatality upon onset of these symptoms [12–15]. Increasing evidence suggests an elevated risk of abnormal blood clotting and thrombosis upon severe infection, including a Kawasaki disease-like syndrome in children, who have been thought to be a low risk age group for disease progression [14, 16].

**Fig. 1:**
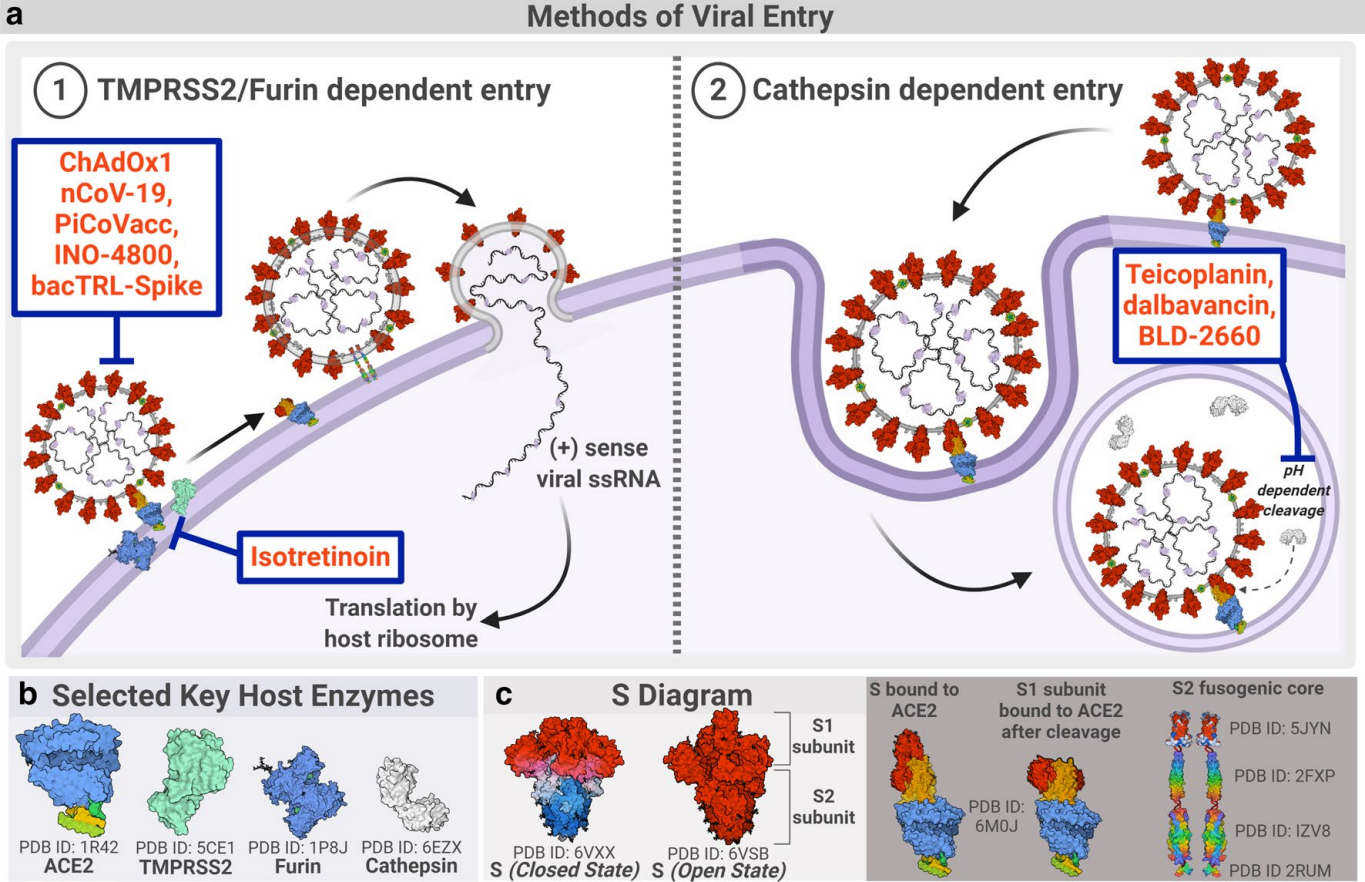
**SARS-CoV-2 Viral Entry Mechanisms and Machinery.** (**a**) SARS-CoV-2 is a lipid membrane, enveloped, plus-sense ( +) single strand (ss) RNA betacoronavirus that must undergo host lipid membrane fusion in order to gain entry into the host cell. Potential inhibitors for subsequent steps of this process are depicted. Enveloped viruses are capable of entering the host cell via (1) direct, neutral pH, plasma membrane fusion or via (2) endocytosis, where membrane fusion would rely on pH-dependent proteases and optimal intra-endosomal conditions [144, 241]. (**b**) Structural diagrams of key enzymes involved in viral-cellular entry. (**c**) Structural diagram of a spike protein (S) depicting the location of S1 and S2 subunits, following S protein cleavage, and the altered conformational states (closed and open). To initiate the entry process, S protein must undergo a conformational change from a closed to open state, which exposes the receptor binding domain (RBD) on S, allowing it to bind to angiotensin converting enzyme 2 (ACE2) on the host cell [40]. Altered S structure bound to ACE2 and S cleaved products are also shown. PDB codes for structures are referenced in Additional file 1: Table 5. Figure was created with BioRender.com

SARS-CoV-2 is a lipid membrane enveloped, plus-sense RNA virus that fuses with the membrane to enter host cells and replicate (Fig. 1) [17]. Infectivity metrics have varied for SARS-CoV-2, depending on region and collection methodology [7, 16]. Estimates suggest the reproduction number (R0), or the expected number of cases directly generated by one individual, was 1.40–3.9 during the initial infection surges in Italy and mainland China, with aggregate measurements calculating the average value to be 2.5–3.5 [16]. The corresponding doubling time has been estimated at 3.1 days for the Italian outbreak—slightly longer than the estimated 1.4–3.0 day doubling time reported in mainland China [16]. By comparison, SARS and MERS boasted estimated R0 and doubling times of 2.0–4.0/2.0–5.0 and 16.2/7–12 days, respectively; these are largely based around isolated datasets and may not represent true values throughout entire populations [18, 19]. The case fatality rates/infection fatality rates (CFR/IFR) estimates for SARS-CoV-2 vary significantly based on age, gender, regional infection prevalence, but current estimates put the absolute rate at approximately 0.68% (0.53–0.82%) [19]. In agreement with several analyses of population-wide outcomes, the largest analysis of SARS-CoV-2 outcomes to date (17.425 million adults), reinforced that age is the predominant risk factor, with the highest hazard ratios (HR) for severe morbidity and mortality following SARS-CoV-2 infection [(Age > 80 + , HR 12.64) vs. (Age > 70, HR 4.77) vs. (Age > 60, HR 2.09)], followed by recent organ transplant (HR 4.27), diagnosis of blood borne malignancy [(< 1 year since diagnosis, HR 3.52) vs. (< 5 years since diagnosis, HR 3.12)], metabolic disease [(uncontrolled diabetes, HR 2.36) vs. (controlled diabetes, HR 1.50) and (obese Class III, HR 2.27) vs. (obese class II, HR 1.56)], male sex (HR 1.99), stroke or dementia (HR 1.79), uncontrolled chronic respiratory conditions (HR 1.78), chronic renal disease (HR 1.72), ethnicity [(Black, HR 1.71) vs. (Mixed, HR 1.64) vs. (Asian, HR 1.62)], as well as other chronic conditions [20]. Multiple epidemiological studies have implicated various micronutrients as potential risk factors for poor disease progression. It remains unclear if such serum values, such as Vitamin C, Vitamin D, Selenium, or Zinc, are directly contributing to poorer outcomes or if these values are a reflection of an acute phase response [21].

SARS-CoV-2 is primarily transmitted by respiratory droplets and aerosols, with relatively less secondary transmission potentially stemming from stable viral particles on surfaces and fomites [22–24]. SARS-CoV-2 has rapidly spread through the community because of its high infectivity rate and asymptomatic viral carriers who unknowingly infect close contacts [25, 26]. Efforts to curb viral spread have differed based on region and municipality, though different methods have been effective at controlling the rate and burden of infection, such as robust testing, contact tracing, self-isolation after confirmed or potential infection, adoption of physical distancing in shared settings, avoidance of large public gatherings, frequent hand washing, use of viricidal disinfectants, and mask wearing [27]. Routine testing protocols followed by aggressive tracing of recent contacts have demonstrated to be effective methods that control viral spread, notably in South Korea and New Zealand [24, 28]. Data continues to emerge regarding the efficacy of maintaining physical distance and mask wearing, especially when indoors, where insufficient ventilation increases the likelihood of viral aerosol transmission and group spread of viral infections [29].

In addition to these methods, advances in SARS-CoV-2 testing have allowed rapid identification of infected individuals. The first generations of tests developed were PCR-based tests pertaining to SARS-CoV-2 specific nucleotide sequences, largely differing only in the primer and probe sequences used by various developers and manufacturers [30, 31]. All first-generation tests were conducted via nasopharyngeal swab, although bronchoalveolar lavage and sputum-based diagnostic tools have since been introduced with comparable efficacy [32]. Each of these methods have been subjected to similar limitations, including the need for a quality primary sample from patients, proper and efficient sample handling, and avoidance of mutations in the viral genome that decrease efficacy of selected primers and probes [33, 34]. More recent generations of PCR-based tests, including less invasive nasopharyngeal and saliva-based tests, are more heat-stable and have less stringent preservation conditions. Even given these limitations, PCR-based in vitro diagnostic (IVD) tools have demonstrated a sensitivity/specificity of 70/90 + % [34–37]. Several of these newer generation tests also demonstrate improved sensitivity and specificity metrics, with values now routinely ranging in the 90/95% + range, respectively [38]. Combining these IVD tools with chest-CT increases the combined sensitivity of diagnosis to up to 94% [35]. Serology-based tests have been implemented; however, these tests have low specificity and positive predictive value. Even at a true population prevalence of 10%, most serology tests do not achieve a positive predictive value above 75%, and many demonstrate a false positive rate of up to 50%. Fortunately, more recent iterations have improved positive predictive value, especially as the population prevalence has increased [39].

Considering the viral genomic, structural, and functional aspects of SARS-CoV-2 and its strains, this review will focus on three precise targets for antiviral activity: spike (S) protein, the main viral protease (M^Pro^), and RNA-dependent RNA polymerase (RdRp). Aspects of these targets will be comprehensively covered, including existing treatment options, challenges to robust and sustained antiviral activity, and potential for modulation and optimization. There is a compelling need for highly effective and rapidly implementable antiviral compounds and therapies, especially because early therapeutic options have shown only minimal capacity to limit morbidity and mortality in the most vulnerable populations. A list of current and emerging therapies is summarized in Table 1 and Additional file 1: Table 1–4.

**Table 1:** Summary of therapies against SARS-CoV-2 targets. Listed are the main viral targets discussed with each drug class

Viral target	Drug classes	Reference table
Spike protein/ACE2 binding	Vaccines, Neutralizing antibodies, ARB and ACE inhibitors, ACE-2 agonist, Fibrosis inhibitor, Hydroxylchloroquine/chloroquine	Additional file 1: Table 1
Main protease (M^Pro^)	HIV protease inhibitors, HCV protease inhibitors, Structural M^Pro^ inhibitors	Additional file 1: Table 2
RNA-dependent RNA polymerase (RdRp)	Nucleoside analogs, Influenza enzyme inhibitors, Zinc supplementation	Additional file 1: Table 3
Whole virus	Inactivated whole virus vaccine, convalescent plasma	Additional file 1: Table 4

### Spike protein pathophysiology

**Fig. 2:**
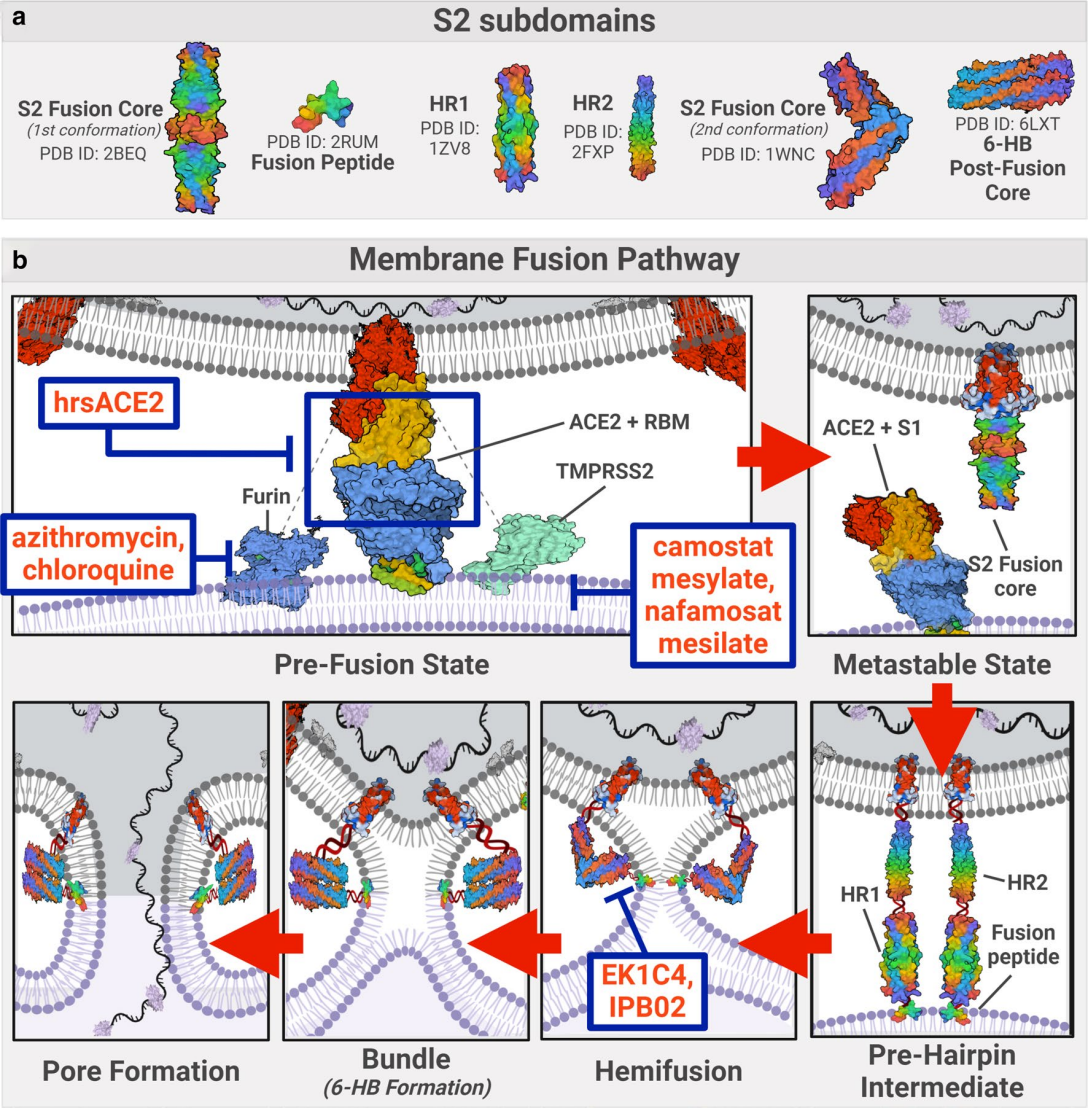
**SARS-CoV-2 Membrane Fusion Pathway.** (**a**) Structural diagrams of some key elements of S2 involved in membrane fusion. (**b**) Schematic summary of the essential steps in viral-host membrane fusion. Following the binding to ACE2, S protein must be cleaved by a protease, such as Transmembrane Serine Protease 2 (TMPRSS2), furin or cathepsin L to generate the S1 and S2 subunits, in order to release the S1 subunit; thus exposing the fusogenic core of S2 [109, 121, 242]. With its hydrophobic core exposed, S2 protein is now in a high-energy, pre-fusion, metastable state, fostered by the energetic imbalance induced by its uncovered core [150]. The S2 subunit can undergo a conformational change, extending heptad repeat 1 (HR1) and heptad repeat 2 (HR2) domains, and injecting its fusion peptide (FP) into the membrane of the host cell, forming the pre-hairpin intermediate. This pre-hairpin structure then folds back into a six helix bundle (6-HB), pulling apart the host membrane. Finally, the viral and host membranes fuse with one another, as HR1 and HR2 fold into a trimer of hairpins resulting in pore formation [152, 243]. The viral genome is then able to access the intracellular space of the host cell for transcription and replication. PDB codes for structures are referenced in Additional file 1: Table 5. Figure was created with  BioRender.com

The S protein is the main virulent and antigenic determinant of SARS-CoV-2 and assembles to form a homotrimeric complex expressed at the external surface of the virus (Fig. 1). This S protein complex protrudes from the virus, peppering the outer lipid membrane like a crown, from which the coronavirus name is derived. It acts to bind its cellular target and to mediate membrane fusion. For SARS-CoV and SARS-CoV-2, angiotensin converting enzyme 2 (ACE2) is the major human receptor for the S protein and facilitates viral entry [3, 40] (Fig. 1). ACE2 is highly expressed in the small and large intestines, kidney epithelium, male gonads, gallbladder, cardiomyocytes, and thyroid follicular cells [41]. More modest expression occurs in respiratory and bronchial epithelium, alveolar macrophages, and type II pneumocytes which may explain why SARS-CoV-2 cases present most commonly as respiratory infections and transmit by aerosols [24, 42]. Collectively, the diversity of expression may contribute to interorgan transmission and systemic manifestations [42]. As previously mentioned, the S protein is cleaved into the S1 subunit, which is primarily responsible for receptor binding, and the S2 subunit, which is involved in the fusion between viral and host membranes (Figs. 1 and 2). Certain conformations are required for each subunit to perform its function, which is why multiple cleavage events are associated with cellular entry [43]. This orchestrated cleavage process is also thought to be important for antigen masking prior to target receptor binding, as immunogenic receptor binding domain epitopes largely remain buried until viral attachment and fusion are initiated [44]. These steps offer several opportunities for therapeutic targeting. The receptor binding domain (RBD) of S protein lies within the S1 subunit and is expressed at the apical surface of each S monomer. Following RBD-ACE2 binding, S1 dissociates from S2 at which point S2 catalyzes membrane fusion (Fig. 2). Potential therapies targeting SARS-CoV-2 S protein will be discussed with emphasis on vaccination, RBD-ACE2 blockade, and fusion inhibitors.

### Vaccines

Vaccination is an attractive therapeutic option as it offers the potential for long-term immunity. The S protein is the logical target for vaccine development because it is expressed at the viral surface and is susceptible to recognition by circulating antibodies. Vaccines designed against S proteins have been most efficacious in vaccine candidates for past betacoronavirus pandemics. Existing strategies for designing an efficacious vaccine include preparations of full-length S Protein, RBD-only peptide, RBD DNA-containing nanoparticles, RBD mRNA-containing nanoparticles, inactivated virus, and recombinant viral vectors. A number of these approaches have proceeded through Phase III clinical trials and will be discussed below.

Moderna’s (Cambridge, MA, USA) lipid nanoparticle mRNA-based vaccine for full length SARS-CoV-2 S protein (mRNA-1273) began Phase III placebo-controlled COVID-19 prevention clinical trials on July 14th, 2020 [45]. Critically, this vaccine candidate demonstrated a vaccine efficacy of 94.1% (94.4% in individuals under 65 with known risk factors, 86.4% in individuals over the age of 65) in its recently completed phase III trial, including robust protection in elderly individuals. The trial enrolled 30,420 individuals (96% of those randomized into the treatment arm received both vaccine injections) that spanned a diverse array of age, socioeconomic, and health demographics, demonstrating virtually complete protection of severe clinical disease and mortality and reporting no sustained adverse events [46].

Notably, this vaccine was awarded $483 million in US federal funding and has partnered with Lonza to produce one billion projected doses annually. Production and orders for this vaccine have escalated significantly in recent months. Clinical trials evaluating the vaccine safety and efficacy in specific populations, including pregnant and youth populations, are now underway. Phase I clinical trials of this vaccine demonstrated robust, dose-dependent neutralizing antibody production and CD4 predominant T cell engagement after administration of two doses of the vaccine, separated by 28 days, both in the age 18–55 cohort and in the age 56 + cohort [47, 48]. Importantly, both B cell and T cell immunity was generated in patients over the age of 71, which represents the most at-risk population for severe COVID-19 outcomes. Vaccination reactions, which were, reportedly, limited to fatigue, chills, headache, myalgia, and pain at the injection site, were reported by over half of recipients when prompted. Synergistic with these findings, recent published data in primates also suggests that mRNA-1273 is able to generate robust T cell immunity, as well as elusive inhibition of mucosal replication in these animals—a limiting factor in multiple vaccine candidates to date [47, 49]. This vaccine requires 2 doses that must be stored at − 20 °C.

BioNTech (Mainz, Germany) partnered with Pfizer to develop four mRNA vaccine preparations encoding either secreted or membrane-anchored full-length or RBD-only S protein constructs (BNT162 b1, b2, b3, and b4) [50]. Of these variations, the b1 (secreted trimerized S  glycoprotein) and b2 (lipid/membrane anchored full-length S protein locked in its pre-fusion conformation) variants emerged as the candidates that entered Phase II/III trials [51, 52]. BNT162b2 was demonstrated to induce relatively fewer and less severe side effects, with equivalent induction of immune response to the b1 variant, and it was therefore chosen to be the construct of choice to be administered for both doses of the recently completed Phase III trial. Similar to the Moderna vaccine candidate, the BNT162b2 demonstrated a vaccine efficacy of 95.0% (94.7% in individuals over the age of 65) among a diverse enrollment of 43,448 individuals. There were no sustained adverse events reported in the experimental group. Trials have begun in additional populations for this vaccine candidate, as well [53]. BioNTech and Pfizer have reported robust immunity induction in a dose-dependent fashion, exceeding SARS-CoV-2 specific antibody titers 1.9–4.6 times greater than those found in convalescent human sera following COVID-19 infection (54). Like mRNA-1273, a significant number of participants reported side effects, the majority of which were also limited to mild-to-moderate flu-like symptoms and pain at the injection site. This vaccine requires 2 doses and must be maintained at − 80 °C.

The University of Oxford, in partnership with AstraZeneca (Cambridge, United Kingdom) also adopted the vectored virus route and have completed several phase III clinical trials involving their candidate vaccine, ChAdOx1 nCoV-19 (Fig. 1a) [54], between April and November of 2020 [55, 56]. The trials demonstrated a collective vaccine efficacy of 62.1% in 23,848 enrolled individuals. Efficacy in older individuals could not be determined from this trial [57]. The trial enrolled individuals across a similarly diverse population distribution, though the trial was marred by incongruencies throughout the trial administration. No lasting long-term side effects could be definitively attributed to the vaccine, though there were at least two cases of transverse myelitis reported in the Phase III trials. Recent evidence suggests similar levels of protection after a single dose, with a booster demonstrating increased serological markers of immunity when given out to 90 days [57]. There are concerns about the possibility of DNA integration from the modified adenoviral vaccine, but this has not been reported to date [58]. ChAdOx1 nCoV-19 is a chimpanzee-derived adenovirus that expresses full-length SARS-CoV-2 S protein. Their published efficacy suggests induction of S-protein-specific neutralizing antibodies in subjects as part of phase I/II trials, as well as in vaccinated rhesus macaques [59, 60]. In pre-clinical development, viral RNA was detected by bronchoalveolar lavage fluid in 33% of vaccinated animals, although this number may be misleading, as viral load was lower in these animals compared to controls, and viral RNA was approaching undetectable levels in almost all vaccinated animals a week after infection. Still, the failure to prevent infection and viral shedding in a third of vaccinated animals raises concerns. Importantly, there was no pulmonary pathology in vaccinated monkeys  seven days post inoculation with SARS-CoV-2, whereas inflammatory infiltrates, hyperplasia, and edema were pronounced in controls [59]. In mice, a booster dose appears to significantly improve vaccine efficacy and protective effects, including in aged mice [55]. Taken together, ChAdOx1 nCoV-19 may prove beneficial by reducing disease severity; however, there is a concern that it may not limit viral spread in the population. Concerns remain over the viability of an adenoviral vaccine delivery mechanism, as a large portion of the population harbors anti-adenoviral antibodies [56].

A recombinant human adenovirus type 5 vaccine developed by CanSino Biologics that expresses full-length S protein has progressed into Stage III clinical Trials [61]. In the phase I trial, 100% of participants in the high dose group (1.5 × 10^11^ viral particles) achieved seroconversion (> fourfold increase in antibody titer) to the RBD at 28 days post-vaccination [62].

Johnson & Johnson’s Ad26.COV2.S is an adenoviral vaccine that has completed Phase III clinical trials and expresses a stabilized pre-fusion S protein complex. Recent releases claim an overall vaccine efficacy of 72% among 43,783 enrolled participants of varied demographics. The vaccine candidate demonstrates an 85% protection from moderate and severe infection, including from the B.1.351 variant. This shot can be easily distributed as a single-shot vaccine and can be stored at normal refrigeration temperatures.

China leads the field in inactivated SARS-CoV-2 vaccine preparations. The most extensively developed of these is sponsored by Sinovac Research and Development Co. Ltd. [63, 64]. Sinovac’s (Beijing, China) purified inactive SARS-CoV-2 virus vaccine, CoronaVac (Fig. 1c), has entered Phase III clinical trials in China, Turkey, and Brazil. The Brazil trial has recently concluded, with preliminary reports claiming an overall vaccine efficacy of 50.4% and a 78% efficacy in prevention. In phase I/II trials, vaccine administration produced robust immune responses in both young and old participants, though immunity was relatively lower in older adults [65]. No severe adverse events were reported, and neutralizing antibodies developed 14 days after the vaccination in its preliminary clinical trial. Similar findings were found after vaccine administration in rhesus macaques [66]. Interestingly, sera of mice vaccinated with inactivated vaccines (as opposed to clonal S protein antigens) display neutralizing efficacy against 11 different SARS-CoV-2 strains with broad phylogenetic variation.

Rapid advances have been made by Novavax (Gaithersburg, MD, USA) in the area of recombinant protein vaccines as the protein subunit vaccine, NVX-CoV2373, has now completed Phase III trials in the United Kingdom. The company’s recombinant S protein vaccine candidate demonstrated an 89.3% vaccine efficacy in over 20,000 enrolled participants and nearly complete elimination of severe disease progression [67]. This included robust protection against the emerging variant strain B1.1.7 (deemed the UK variant); however, vaccine efficacy dropped to 60% in preventing the B.1.351 variant (South African variant). In pre-clinical and early phase trials, NVX-CoV2373 induced robust anti-S protein antibody titers as well as CD4 + T helper cell reactivity. Novavax has received a $1.6 billion investment from the United States Warp Speed project with intent to produce 100 million doses of the NVX-CoV2373 candidate vaccine [68].

The first DNA vaccine, INO-4800 (Fig. 1a), is currently in a phase I/II trials [69]. Inovio (Plymouth Meeting, PA, USA) developed a vaccine encoding full-length S protein induced T cell responses and S1, S2, and RBD-specific IgG production in mice. Markedly, sera of INO-4800-vaccinated mice inhibited ACE2 binding for S protein, which would offer the added benefit of limiting viral spread [70]. Inovio announced in a press release that 94% of vaccine recipients produced threshold immune responses, noting a robust induction of both neutralizing-antibody and T cell response in these recipients. Inovio reports that it plans to continue Phase III trials in early 2021 after being paused by the US FDA pending further investigation. Separately, a comparable strategy developed by Symvivo (British Columbia, Canada) employs *Bifidobacterium longum* engineered to deliver a DNA plasmid encoding the full-length SARS-CoV-2 S protein (SARS-2-SP). The vaccine, named bacTRL-S (Fig. 1a), has initiated phase I clinical trials in British Columbia and Nova Scotia, Canada [71]. While no preclinical data is available, theoretically, the pathogen-associated molecular patterns (PAMPs) of the bacterial vector could help boost an adaptive immune response.

A significant number of additional vaccine and antibody-based therapies are in various stages of pre-clinical and clinical developments (Table 1 and Additional file 1: Table 1–4) [72–74].

### RBD-ACE2 blockers

While vaccination is an ideal modality for SARS-CoV-2 prophylaxis, achieving neutralizing antibody titers high enough to prevent infection can take weeks [75]. It is important to have therapies available to treat patients infected with SARS-CoV-2 before a vaccine is readily available to everyone and can be potentially useful in different strains when vaccines are less effective. Treatments that precisely inhibit RBD-ACE2 interactions may play an important role in reducing morbidity and mortality as this receptor-ligand interaction is essential for host cell entry [3, 76]. Vaccine-derived antibodies overlap with this strategy since RBD has been identified as the predominant antigen targeted by vaccine-induced antibodies against the S protein [66]. For this reason, viral neutralizing antibodies are commonly presumed to be RBD-specific. However, this is not always the case, as antibodies binding S outside the RBD have neutralizing efficacy without inhibiting ACE2 binding [77, 78]. Similarly, RBD-binding antibodies can neutralize viral particles without competing for ACE2 binding [79, 80]. It has been reported that destabilization of the prefusion metastable complex by antibody binding can disrupt virulence in the absence of competition for the ACE2 binding site [81]. To our knowledge, no study has compared the neutralizing efficacy of antibodies that do or do not competitively antagonize RBD-ACE2 interactions. This information could potentially narrow the search for the optimal monoclonal anti-SARS-CoV2-S antibody. Over 160 clinical trials examining convalescent plasma for SARS-CoV-2 treatment are accessible on Clinicaltrials.gov. It is possible that a polyclonal repertoire of IgG/IgM clones obtained in plasma may synergize mechanistically and provide greater efficacy than monoclonal strategies. Indeed, convalescent sera therapies will likely be less susceptible to treatment resistance as new SARS-CoV-2 strains evolve. Detailed discussions of the clinical efficacy of convalescent sera can be found in a recent review [82]. Among the monoclonal antibodies that have progressed through Phase III clinical trials, only Regeneron’s REGN-COV2 has demonstrated apparent efficacy throughout Phase I/II clinical trials. The REGN-COV2 cocktail (since renamed REGEN-COV, asirivimab and imdevimab), consisting of two fully humanized monoclonal antibodies against the SARS-CoV-2 S protein, reduces viral load in proportion to initial viral load at the onset of treatment, and Regeneron has announced a 100% reduction in severe disease in individuals receiving the drug cocktail. The antibody cocktail binds and sequesters SARS-CoV-2 viral particles, preventing their interaction with cellular receptor proteins [83, 84].

Unfortunately, any discussion of S protein targeting therapies is incomplete without a discussion of emerging strain variations and genetic variability. Since the time the SARS-CoV-2 genome was first sequenced in January 2020, many mutations have been identified in samples isolated from patients in similar locations, indicating the virus may diverge into several sub-strains [6, 7, 85, 86]. This is relevant to drug and vaccine design, since testing efficacy, antiviral resistance, and vaccine efficacy may depend significantly on the genetic stability of target epitopes. At least 93 distinct mutations have been isolated from different regions, with the largest percentage clustered in the open reading frame 1b (ORF1b) (48 mutations) and the S protein (14 mutations) encoding regions. Of these mutations, a number of definitive lineages have emerged [87]. Perhaps most prominent among these are B.1.1.7 (UK variant), B.1.351 (South African variant), and the P.1 (Brazil, B.1.1.28 branch) lineage [87]. The branch lineages predominantly represent alterations in the immunoreactive regions of the S protein. These variants have demonstrated varying degrees of immune escape, including from convalescent sera [88].Vaccine efficacy against these variants has been variable, with almost all approved candidates retaining efficacy against the B.1.1.7 (UK) variant. Efficacy has been less consistently retained in the B.1.351 variant, including significant reductions in efficacy seen in the Johnson and Johnson, NovaVax, and Oxford/AstraZeneca vaccine [89, 90].The exact efficacy of each of these vaccines will be clarified by additional data. Fortunately, none of the variants have demonstrated apparent differences in virulence and mortality. Multiple variants initially appeared to be more infectious; however, recent variant cases have reduced that challenge this claim—namely whether they are truly more infectious or were solely novel pathogens in affected regions. The variant regions are also common targets for diagnostic tools and therapies, making both the frequency and the location of mutations directly relevant to the efficacy of viral treatment and containment [6, 7, 85, 86]. Specifically, PCR-based IVD technologies use primers that are commonly complementary to regions in the ORF1 or S protein sequence. Several antivirals target charge-specific locations in either the RdRp/nonstructural protein (nsp) 12, receptor binding domain, viral proteases, or viral-activating/processing enzymes, either at the nucleic acid or protein level. Of the virus-targeting therapeutics that have been developed or in pre-clinical development (Table 1), nucleoside analogs and phagolysosome modulators are potentially more resistant to genetic mutations. Many other treatments could be influenced by changes in viral structure and are more susceptible to viral mutations.

Reducing the expression of cellular ACE2 offers a separate strategy for limiting viral infection. There was initial concern that patients taking ACE inhibitors and angiotensin II receptor blockers (ARBs), which are known to upregulate ACE2 expression, would increase infection susceptibility [91]. However, no evidence has emerged suggesting that renin–angiotensin–aldosterone system (RAAS) inhibitors negatively impact patient outcomes [92–94]. In fact, these agents might actually improve clinical course for hospitalized SARS-CoV-2 patients [95]. Although these data need to be confirmed, the present evidence would suggest that RAAS inhibitors should not be stopped in the setting of SARS-CoV-2. Conversely, it has been hypothesized that downregulating ACE2 would reduce viral infection and improve outcomes. Isotretinoin (Fig. 1a), an FDA-approved acne medication, was predicted to be the strongest down-regulator of ACE2 expression [96]. Several trials have incorporated isotretinoin into trials to treat SARS-CoV-2 alone or as a combination therapy to enhance other RBD-ACE2 targeting agents [97–101]. It should be noted that the immunomodulatory effects of isotretinoin may improve outcomes independently of its ACE2-regulating effects, however these mechanisms are outside the scope of this review.

Non-antibody therapies targeting the RBD-ACE2 axis are more simplistic mechanistically in that they are exclusively competitive antagonists and steric inhibitors of target engagement. Soluble SARS-CoV-2 RBD inhibited pseudoviral entry in human ACE2-expressing cells [102]. In the setting of acute therapy, existing antibodies against SARS-CoV2 RBD may inhibit its efficacy. There are currently no clinical trials investigating recombinant RBD peptides for treating SARS-CoV-2, however the preclinical groundwork has been laid for therapeutic development [103]. In contrast, two clinical trials forms utilizing recombinant ACE2 protein to treat SARS-CoV-2 have been approved for enrollment [97, 104]. Before, the COVID-19 pandemic, Khan and colleagues found human recombinant soluble ACE2 (hrsACE2) was well tolerated by patients receiving treatment for acute respiratory distress syndrome (ARDS) [105]. Preclinical work investigating hrsACE2 in the setting of SARS-CoV-2 found that hrsACE2 inhibited viral attachment and replication within ACE2-expressing Vero E6 cells (Fig. 2b) [106]. It has also been reported that L-SIGN and DC-SIGN are low affinity receptors that can mediate SARS infection [107, 108]. Limited work has been done to develop therapeutics specifically targeting these alternative entry mechanisms. As this is the third coronavirus outbreak since 2002, the probability of another outbreak is almost certain. Of these outbreaks, SARS-CoV and SARS-CoV-2 both facilitate endosome-mediated infection by binding ACE2 [3, 40, 76]. Developing novel therapies that target RBD-ACE2 interactions will likely benefit patients affected by SARS-CoV-2. Importantly, these therapies could be rapidly adapted to treat future coronavirus serotypes that target ACE2.

### Viral membrane fusion inhibitors

The ACE2 binding of S protein induces a conformational change that opens cleavage sites accessible to cellular proteases (Figs. 1 and 2). Cleavage at the S1/S2 and then S2′ sites induce conformational changes that permit the catalytic fusion of viral and cellular membranes by the fusion protein [109] (Figs. 1 and 2). Inhibiting select proteases, such as cathepsins, furin, and transmembrane protease serine 2 (TMPRSS2), offers alternative methods to prevent viral-host membrane fusion, thus halting its invasion. Preclinical data investigating TMPRSS2 has shown promise as a therapeutic target. Camostat mesylate, a serine protease inhibitor, can perturb TMPRSS2 activity which is vital for viral entry and replication within Calu-3 (lung epithelial) cells (Fig. 2b) [110]. Although the greatest effect was achieved by targeting TMPRSS2, the authors noted that the degree of viral inhibition was increased with the concomitant use of a cathepsin inhibitor. Oral camostat mesylate and the more potent intravenous serine inhibitor, nafamosat mesilate (Fig. 2b), are approved treatments for pancreatitis in Japan [111]. Nafamosat mesylate is currently under investigation in Phase II/III trials (NCT04418128). The satisfactory safety profiles associated with these drugs has permitted the rapid enrollment of patients for phase II and III clinical trials [112–115].

Precise targeting of cathepsins, a class of cysteine proteases, may improve the efficacy of TMPRSS2-directed interventions as suggested in preclinical models [110]. Indeed, it has been suggested that cathepsin L (CatL) is vital for facilitating S protein-directed entry into HEK293T cells [76]. Teicoplanin and dalbavancin (Fig. 1a), two glycopeptide antibiotics, could prevent S-directed pseudoviral entry in vitro by inhibiting CatL [116]. The calpain and cathepsin inhibitor, BLD-2660 (Fig. 1a), is a small molecule that was initially designed for fibrotic diseases but is currently being adapted for SARS-CoV-2 patients. The anti-IL-6 and anti-fibrotic actions paired with the hypothetical benefits of cathepsin inhibition make BLD-2660 an attractive candidate for treatment [117]. However, accumulating evidence suggest that these effects may be mitigated by anti-inflammatory effects. This is supported by the relative efficacy of corticosteroids (namely dexamethasone) and anti-IL-6 modalities (namely Tocalizumab), as implementation of dexamethasone has decreased mortality by as much as 35% in severe patients in some studies. Dexamethasone is now considered as a component of the standard of care in treating moderate to severe disease, while recent evidence also suggests that Tocalizumab may convey clinically relevant efficacy in preventing disease progression and sequelae [118–120].

Furin and furin-like proprotein proteases are ubiquitously expressed and have dynamic functions. Coutard and others predicted furin cleavage sites unique to SARS-CoV-2 within the S1/S2 and S2′ domains [121]. Hoffman and others extended this work to find that furin cleaves SARS-CoV2-S protein at the S1/S2 motif, and that this cleavage is essential for viral entry into human lung epithelium and cell–cell spread [109, 122]. While no registered clinical trial targeting furin for the treatment of SARS-CoV-2 exists, one study is designed to investigate the role of tranexamic acid, a plasmin inhibitor, in the cleavage of the S protein complex at the furin site [123]. This may change as the understanding of S protein cleavage advances, but human therapies will likely need to be short in duration and aerosolized to prevent undesirable systemic toxicities. Interestingly, preprint data from Poschet and others report azithromycin and chloroquine (CQ) reduce furin activity, which might derive from their putative lysosomotropic actions (Fig. 2b) [124]. TMPRSS2 is the most rational precision target because it boasts the greatest reduction in SARS-CoV-2 virulence while maintaining low toxicity.

S protein proteolytic cleavage can also be inhibited by endosomal pH alterations [110, 125]. CQ was shown to inhibit the acidification of endosomes which prevented SARS-CoV-SP-mediated pseudovirus infection in Vero E6 cells [125]. Vincent and others extended this work to find that CQ impaired glycosylation of ACE2, which may also impair viral infection [126]. Given that SARS-CoV-2 utilizes ACE2 to undergo receptor-mediated endocytosis, it is plausible that the anti-viral mechanisms would translate in to SARS-CoV-2 [76]. In a letter to the editor, Wang and colleagues report that CQ inhibited SARS-CoV-2 infection and replication in Vero E6 cells which would support the proposed mechanism of action [127]. CQ and its metabolite, hydroxychloroquine (HCQ), are FDA-approved drugs for autoimmune and parasitic conditions which has spurred its rapid incorporation into clinical trials and off label use. Initially, CQ came under criticism after a 96,032 patient cohort study reported increased mortality associated with SARS-CoV-2 patients receiving CQ/HCQ in the presence or absence of a macrolide. This paper has since been retracted after the validity and rigor of the multinational registry used to acquire the data could not be verified [128]. Since, several large datasets have emerged that illustrate that HCQ is ineffective in reducing either morbidity or morality secondary to COVID-19 infection [129–134] Presently, clinical outcomes do not appear to improve with CQ/HCQ treatment, with or without adjuvant treatment [135–138].

Thus far, the pharmaceuticals discussed have interfered with S protein cleavage which is required for virus-cell fusion. Another putative therapeutic approach could be to directly interfere with the fusion motifs located on S2. Following RBD-ACE2 engagement and receptor-mediated endocytosis, S1 is cleaved and released form S2 which permits the insertion of the fusion peptide (FP) into the endosomal membrane [109, 139, 140]. FP insertion induces the helical heptad repeat 1 (HR1) motif to self-assemble into a trimeric coiled-coil structure. Next, S2 folds in upon itself whereby the distal HR2 helices insert into the grooves of the apical HR1 coil forming a stable 6 helix bundle (6-HB) [141–145]. S2 folding and formation of the 6-HB shortens the distance between viral and host membranes (Fig. 2). This juxtaposition causes the viral and endosomal lipid bilayers to fuse with one another so that the virion can escape the endosome and access the host cytoplasm [146–150]. SARS-CoV-2 is unique from SARS-CoV in that it can induce syncytial formation by catalyzing cell–cell fusion [141]. Therefore, therapies targeting the S2 fusion machinery have the potential to act at the level of endosome-cytoplasmic entry as well as spread between adjacent host cells. Recombinant HR1/HR2 peptides can disrupt 6-HB formation and prevent membrane fusion. There are two known compounds, EK1 and IPB01, which are HR2 sequence-derived peptide that prevent 6-HB formation by binding to HR1 [151, 152]. Attaching carboxy-terminal cholesterol groups to each peptide enhanced the antiviral efficacy and potency of these compounds. The updated lipoprotein names for the aforementioned peptides are EK1C4 and IPB02, respectively (Fig. 2b) [141, 152]. There are no studies registered for the use of HR-targeting compounds, but development should be encouraged as such compounds already display reactivity against a broad range of coronavirus strains.

### Pro-teasing out the main protease (M^Pro^) and its inhibitors

**Fig. 3:**
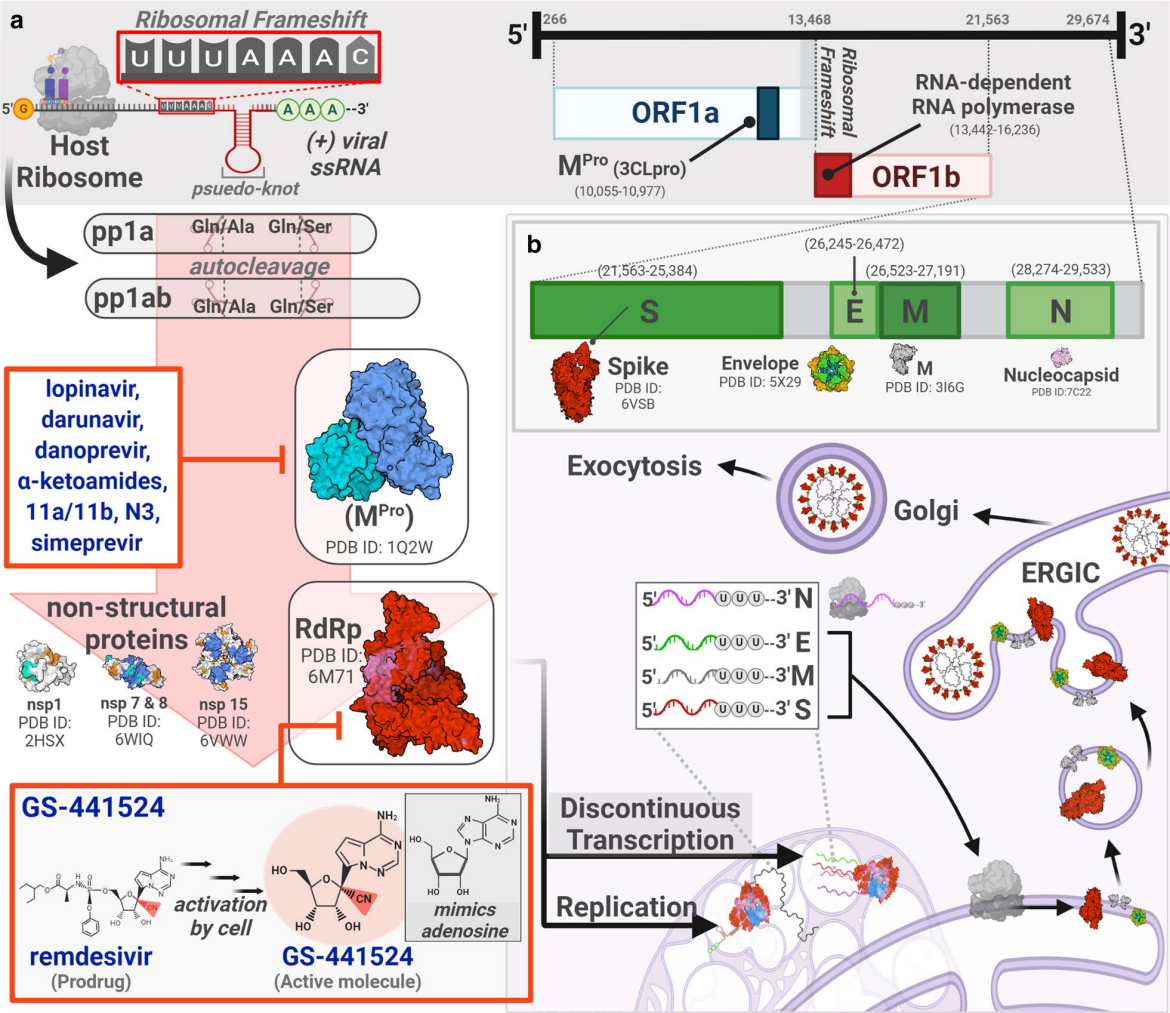
**Key elements of SARS-CoV-2 viral replication and some therapeutic targets.** (**a**) ( +) RNA viruses are ‘ribosome-ready’, meaning that upon cytoplasmic entry, their genome is recognized by the host ribosome as mRNA and can immediately be translated. During translation, the viral genome employs a technique called 'ribosomal frameshifting' to produce two types of polyproteins, pp1a and pp1ab, which encode the non-structural proteins (nsps) including the viral protease M^Pro^/nsp5 [232]. M^Pro^ first autocleaves  the polyproteins at a Gln/Ala and Gln/Ser junction, then cleaves most of the remaining proteins from the first two reading frames of the viral genome, including RdRp [232]. RdRp integrates with nsp7 and nsp8 to assemble into the polymerase holoenzyme. (**b**) The 3′ region of SARS-CoV-2 RNA genome encodes its structural proteins, S, Envelope (E), Membrane (M) and Nucleocapsid (N) proteins. Discontinuous transcription of the 3′ region generates a nested set of subgenomic ( −) RNAs that are copied into ( +) mRNA, resulting in the host ribosomal translation of the structural proteins [196]. RdRp is also responsible for replicating the viral genome for packaging. Replication and transcription processes are localized into interconnected, double-membraned, ER-derived vesicles called replicase-transcriptase complex (RTC) [189, 244], which centralize the machinery required for these processes and serve as a buffer to any host immune response [245]. Viral structural proteins are translated by host ribosomes from the subgenomic RNA synthesized by RdRp. After processing in the ER-Golgi Intermediate Compartment (ERGIC), the structural proteins and viral RNA are transported to budding vesicles. Finally, virus particles are assembled and released by exocytosis. PDB codes for structures are referenced in Additional file 1: Table 5. Figure was created with BioRender.com

In coronaviruses, the main protease (M^Pro^), also known as 3C-like proteinase (3CLpro) or nsp5, performs the first major step to activate viral replication [153–155]. M^Pro^ is encoded in two large polyproteins, pp1a and pp1ab (Fig. 3), which are cleaved by autoproteolysis to release a series of nonstructural proteins (nsps) involved in viral replication [156, 157]. This initial cleavage step performed by M^Pro^ is a necessary first step before it can activate other proteins involved in coronavirus replication. M^pPro^ consists of three major domains containing a Cys-His catalytic dyad and a group of four major substrate-binding sites located between domains I and III (often referred to as S1, S2, S3, and S4 sites) [158–161]. Overall, the SARS-CoV-2 M^Pro^ shares 96% of its DNA sequence identity with the SARS-CoV M^Pro^ and multiple domains, such as the substrate-binding sites, are well conserved among many coronaviruses [162, 163]. M^Pro^ acts on 11 highly specific cleavage recognition sites of Leu-Gln↓(Ser, Ala, Gly) between different interdomain junctions [153, 155, 158]. The M^Pro^ structure has low homology with endogenous human proteases, which makes it an ideal target for highly specific protease inhibitors with low toxicity [158]. By inhibiting the action of M^Pro^, it is possible to prevent the activation of other proteins required for viral replication.

M^Pro^ substrate binding relies on a conserved residue pair of Glu-His to sterically recognize Gln residue on the target substrate in some coronaviruses [164]. This recognition depends primarily on Gln’s carbonyl group, thus potential M^Pro^ inhibitory compounds should mimic Gln side chain volume, rather than focus on its electrostatic components [153, 162, 164]. M^Pro^ catalysis also relies on a conserved GSCGS motif that maintains the structure of its triple turn catalytic site, located directly opposite of a stabilizing region, partial negative charge cluster (PNCC). In various coronaviruses, PNCC interacts with a water residue to stabilize Turn II of the active site increasing the efficiency of M^Pro^ catalytic activity [164]. Since PNCC is located on the enzyme surface, it is a potential target for allosteric inhibition [165, 166].

In SARS-CoV-2, M^Pro^ can have other secondary functions and was found to interact with histone deacetylase 2 (HDAC2), which has a potential cleavage site near the nuclear localization sequence [167]. This suggests M^Pro^ could interrupt the nuclear transport of HDAC2 and inhibit its effects on inflammation, resulting in an overall anti-inflammatory effect. Direct inhibition of M^Pro^ would interfere with the replication cycle and its other functions at limiting inflammation. Given the important roles of M^Pro^, protease inhibitors that directly target its unique structure could prove to be effective and form a major component of a drug cocktail to limit SARS-CoV-2 infections (Table 1 and Additional file 1: Table 2).

Many protease inhibitors are in preclinical development and could become an invaluable tool to directly inhibit the main protease and disrupt the primary viral lifecycle. Many of these compounds are peptidomimetic drugs screened by computational modeling, in vitro assays, or cell-based assays. Some drugs were previously effective in other coronaviruses such as SARS-CoV or MERS. Based on computational modeling and a high throughput screening, N3 compound was found to be an irreversible protease inhibitor that can covalently bind to the active site of M^Pro^ and block the entry and docking of other substrate molecules (Fig. 3a) [168]. This tight covalent interaction was confirmed by crystal structure models showing a dimer complex of N3 and M^Pro^, further stabilized by multiple hydrogen bonds to fully anchor it. N3 was found to be effective at inhibiting M^Pro^ in vitro and in limiting SARS-CoV-2 infection in Vero cells [168].

To specifically target M^Pro^, Compounds 11a and 11b were another group of peptidomimetic drugs designed to interact with the substrate binding sites of M^Pro^, which directly inhibits its catalytic activity (Fig. 3a) [165]. 11a and 11b have aldehyde groups that can covalently bind to a cysteine in M^Pro^, and this interaction is further stabilized by additional hydrogen bonds and other interactions. 11a and 11b have shown high efficacy in inhibiting the main protease in vitro. Promisingly, both compounds can be administered intravenously with low toxicity in mammals such as rats and dogs and will potentially be safe in humans. α-ketoamides are another class of structural protease inhibitors that can target the substrate binding sites of M^Pro^ and block its proteolytic activity (Fig. 3a) [158, 169]. In particular, α-ketoamide 13b inhibited the proteolytic activity of recombinant SARS-CoV-2 and MERS M^Pro^ and significantly limited SARS-CoV-2 replication in Calu-3 lung cells*.* These compounds can be well tolerated and delivered by subcutaneous administration or lung inhalation due to lung tropism.

Previously shown to be effective against SARS-CoV-1, compounds such as compound 4, GC376, MAC-5576 could also potentially target the SARS-CoV-2 M^Pro^ [170]. In cell-based assays, compound 4 and GC376 significantly inhibited viral replication and was safe enough to not induce cytotoxicity. These compounds can covalently bind to the active Cys145 residue of the substrate-binding site of M^Pro^ with different modifications such as by nucleophilic addition in the case of GC376. Out of the four major substrate-binding sites, these inhibitors seem to primarily target the first or second sites (S1 or S2) more than the third and fourth sites (S3 or S4) [170]. Further development of structural protease inhibitors could improve inhibition of all four substrate-binding sites or be used in combination to simultaneously target multiple substrate-binding sites. In addition to the active site, other allosteric sites such as the dimer surface or distal site regions can also be targeted to modulate and inhibit the catalytic activity of M^Pro^ [171]. A combination of different protease inhibitors against both the substrate-binding site and distant allosteric sites could be used synergistically to enhance M^Pro^ inhibition and completely stop viral replication.

While protease inhibitors are primarily in preclinical development, early results show that they are very promising and could potentially be effective at preventing and limiting SARS-CoV-2 infections. Protease inhibitors would be useful in patients with early or moderate symptoms, or possibly for prophylaxis to completely limit early spread and replication. This will also be a good option for patients who are immunocompromised or have other contraindications and cannot directly receive a vaccine. In addition to its effectiveness, protease inhibitors may also have low toxicity and fewer contraindications, because SARS-CoV-2 M^Pro^ does not share a lot of homology with existing human proteins [158]. This will also decrease the chance for severe side effects and allow more frequent use in different patient populations. Additional modifications can help allow targeted delivery of protease inhibitors into infected cells in the lungs. Some of these compounds, such as α-ketoamides, can be administered by inhalation directly to the lungs, as opposed to intravenous injection, which can further improve its effectiveness [158, 172]. Future studies should test protease inhibitors in preclinical animal models and evaluate them further in clinical trials.

### Repurposing HIV and HCV protease inhibitors

Existing protease inhibitors against other viruses such as HIV (human immunodeficiency virus) and HCV (Hepatitis C virus) were considered for use in COVID-19 because they could nonspecifically target viral proteases in general. They are existing FDA-approved drugs with good safety profiles and can be easily mass produced and distributed to many patients, if shown to be effective against SARS-CoV-2. Lopinavir (Fig. 3a) is an aspartate protease inhibitor used in HIV treatment and is often administered with ritonavir to increase its half-life by inhibiting its degradation by cytochrome P450 3A4 [173]. The combination of lopinavir and ritonavir was effective in in vitro studies and trials of patients with SARS-CoV and suggested that this combination could potentially inhibit the SARS-CoV-2 main protease and viral replication [174, 175]. Lopinavir/ritonavir does not appear to cause any serious side effects or complications. In early July of 2020, the multi-national World Health Organization (WHO) Solidarity Trial paused the recruitment and study of its lopinavir/ritonavir treatment arm for hospitalized patients with severe symptoms due to futility [134, 175, 176]. Data to date suggests that lopinavir/ritonavir does not significantly alter the mortality rate when compared to control groups that received supportive care [134, 177]. There may have also been adverse side effects in some patients that supported the decision to stop the use of lopinavir/ritonavir. Similarly, the lopinavir/ritonavir arm of the Randomised Evaluation of COVid-19 thERapY (RECOVERY) Trial headed by the University of Oxford in the United Kingdom did not have a significant improvement in mortality rate or a reduction in hospital stay for almost 1600 hospitalized patients, many of whom were on supplemental oxygen [176, 178]. However, it was noted that lopinavir/ritonavir was not completely evaluated in patients with mechanical ventilators due to the smaller sample size for this subgroup. Results from both the WHO Solidarity Trial and the RECOVERY Trial suggested that lopinavir/ritonavir may be ineffective at treating a large sample size of hospitalized patients with SARS-CoV-2 infection and did not have a significant reduction in the mortality rate or other measured clinical outcomes.

Darunavir is another HIV protease inhibitor that was considered for use in COVID-19, because its antiviral activity was similar to lopinavir. Like the interaction between lopinavir and ritonavir, cobicistat is often given with darunavir to inhibit cytochrome P450 3A4 activity and increase the bioavailability of darunavir. In vitro studies, however, showed that darunavir did not restrict SARS-CoV-2 infection of Caco-2 cells or improve cell viability [179]. An initial pilot study assessed the effects of darunavir/cobicistat on viral clearance in COVID-19 patients with milder symptoms that recently tested positive [180]. Compared to standard care, five days of darunavir/cobicistat did not significantly reduce viral load or affect the time to a negative test over a week, but none of the study participants had serious side effects. Other clinical trials involving darunavir/cobicistat are ongoing and a larger sample size could better determine its effectiveness. Similarly, ASC-09, a modified structural version of darunavir, is also being evaluated in combination with ritonavir in different clinical studies [181, 182].

In addition to HIV protease inhibitors, HCV protease inhibitors may also have inhibitory action against M^Pro^, since HCV NS3 proteases have structural similarities with M^Pro^ [183]. Recent in vitro screening of HCV protease inhibitors found that simeprevir has been shown to effectively inhibit SARS-CoV-2 replication in cell-based assays using Vero E6 cells and human HEK293T cells [183–185]. In addition to inhibition of M^pro^, simeprevir may also have inhibitory action on RdRp[185] and can be used synergistically with other drugs such as remdesivir [183–185]. Since simeprevir is a FDA-approved drug, it could be quickly tested in clinical trials.

Danoprevir is another repurposed HCV protease inhibitor and is often given with ritonavir to inhibit cytochrome P450 3A4. The first reported clinical study gave treatments of danoprevir/ritonavir to 11 patients with moderate symptoms from COVID-19 [186]. 4 to 14 days of treatment with danoprevir/ritonavir helped all the study participants recover and be discharged from the hospital without any major side effects. However, it is difficult to fully assess if danoprevir/ritonavir is effective at reducing viral load without an adequate comparison to proper control groups. Another HCV serine protease inhibitor is boceprevir, which was screened to interact with M^Pro^. Boceprevir inhibited recombinant M^Pro^ enzymatic activity and inhibited cytopathic effects of SARS-CoV-2 in Vero cells [187]. While these initial results are promising, future studies should fully evaluate the antiviral effects of HCV protease inhibitors such as danoprevir/ritonavir and boceprevir in various animal models and randomized clinical trials.

Overall, the clinical trial results with repurposed HIV protease inhibitors such as lopinavir/ritonavir and darunavir/cobicistat for COVID-19 have been underwhelming and showed no significant effects on mortality rate, length of hospital stay, or other outcomes. Several factors could explain the recent failure of HIV protease inhibitors in different clinical trials. Some of these studies focused on using lopinavir/ritonavir in hospitalized patients with severe symptoms who required supplemental oxygen or relied on mechanical ventilator support. Protease inhibitors are likely most effective to limit replication in early stages of disease and may not have a significant impact in severe infections when viral load is extremely high and cannot be fully mitigated. HIV protease inhibitors may also be insufficient by itself and require the synergistic addition of other therapies to enhance its efficacy. In a preliminary clinical study, lopinavir/ritonavir combined with interferon beta, an immune modulator, and ribavirin, a nucleoside analog and inhibitor, improved symptoms and decreased infection time compared to lopinavir/ritonavir by itself and to standard care [188]. These findings were not replicated in larger patient cohorts within the SOLIDARITY trial, however. Another possibility is that HIV protease inhibitors do not sufficiently bind to the active site or regulatory domains to fully inhibit the activity of M^Pro^ in SARS-CoV-2 for a significant clinical effect. It appears that repurposing protease inhibitors that target other viruses outside of coronaviruses may be insufficient for SARS-CoV-2. Thus, specific protease inhibitors designed to target the SARS-CoV-2 M^Pro^ with higher affinity may be more effective and are more likely to show clinical improvement in patients.

### RNA dependent RNA polymerase

RdRp or nsp12 is the viral enzyme responsible for both replicating the RNA genome and transcibing the RNA used for translating the structural and accessory proteins at the 3′ end of the genome. Both of these events occur in interconnected double membrane vesicles that bud off of the host cell’s ER, called replication and transcription complexes [189].

For genomic replication, polymerases employ a conserved method of nucleic acid polymerization called the two-metal mechanism of polymerase catalysis. RdRp catalyzes the formation of a phosphodiester bond using metal ions that are held in place by two conserved aspartic acids in its active site. The conserved sequence for a ( +) strand RNA polymerase like the one found in SARS-CoV-2 is a Gly-Asp-Asp motif. This motif is similar to all other polymerases suggesting a common evolutionary ancestor.

Resulting in the translation of the 3′ end of the viral genome, RdRp employs an unusual strategy of discontinuous transcription producing a nested set of 3′ co-terminal sub-genomic RNAs. As RdRp copies the viral RNA, it reaches junctions called Transcription Regulatory Sequences (TRS) which contain highly conserved Core Sequences (CS). Once these sequences are detected by RdRp, it is able to either copy the sequence or jump from that sequence, possibly through long-range RNA-RNA interaction, and base pair with same CS part of the TRS at 5′ end of genomic RNA resulting in the production of (−) RNAs. RdRp then copies these (-) subgenomic RNA sequences into ribosome ready mRNA. The complicated nature of discontinuous transcription may help explain the higher rate of recombination seen in coronaviruses [190]. RdRp also complexes with nsp7 and nsp8 which help to increase RdRp processivity [165, 191–193], and interacts with nsp14—a bifunctional protein that has capping and endonuclease activities [194].

Due to its importance in viral replication, RdRp has been the target of many anti-viral therapies and inhibition of the polymerase may be an effective method of reducing SARS-CoV-2 transmission and disease severity (Table 1 and Additional file 1: Table 3). RdRp inhibitors have been studied and successfully used in the past to manage a myriad of diseases with viral etiologies including HIV, Hepatitis C, and Ebola [191, 195–197]. There is also data on the use of RdRp inhibitors for treatment of SARS-CoV and MERS infections, which are genetically and structurally similar to the SARS-CoV-2 virus [198]. Currently, there has been only one missense mutation in the viral RdRp found in the top 50 most common mutations in SARS-CoV-2 across the globe [199]. This indicates that the SARS-CoV-2 RdRp is conserved, which decreases the risk of viral resistance to an RdRp inhibitor. Recent cryo-electron microscopy research has elucidated the structure of the SARS-CoV-2 RdRp revealing that it retains the typical ‘hand’ formation common to polymerases; its structure comprises of the fingers, thumb, and palm subdomains. This commonality allows researchers to use information from previous RdRp inhibitor studies as a foundation to jumpstart their experiments with new data. Characterization of the SARS-CoV-2 RdRp provides a framework for repurposing previously used drugs and developing new medications to inactivate the SARS-CoV-2 virus.

### Nucleoside analog RdRp inhibitors

Antiviral nucleoside analogs are prodrugs that are converted into the active 5-triphosphate form within a cell. This nucleoside analog is then incorporated by viral RNA polymerase into viral RNA strands leading to termination of RNA polymerase function or becoming incorporated into a complete viral RNA strand but leading to non-functional mutations. These mechanisms of action are not mutually exclusive and often both contribute to decreased viral load. Coronaviruses are known to have an exonuclease (nsp14) with proofreading activity, which can remove incorrectly paired nucleotide bases and lead to resistance against nucleoside analogs [200, 201]. Yet, some drugs are still effective including remdesivir which mainly works by terminating the viral RdRp (Fig. 3a) [202]. Recent data, largely stemming from the SOLIDARITY trial, suggests that remdesivir provides very minimal benefit in terms of morbidity and mortality in the context of a well-controlled clinical trial [134].

In silico assays, which use computer models to predict a molecule’s affinity to an enzyme, and molecular docking studies have illustrated that many drugs that have been used to treat various diseases and a myriad of biologically derived compounds can bind to SARS-CoV-2′s RdRp. These molecules provide a potential starting point for SARS-CoV-2 treatment, but none have been proven effective and most are far from becoming a therapeutic option. [195, 203–207]. Furthermore, molecular analysis studies have been completed to show the binding site and molecular mechanism of action of remdesivir on the SARS-CoV-2 RdRp [191, 208]. In vitro cell assays of SARS-CoV-2 infection models tested the effectiveness of known HIV nucleoside analogs including tenofovir, 40-ethynyl-2-fluoro-20-deoxyadenosine, alovudine, lamivudine, and emtricitabine as well as remdesivir to inhibit viral loads and discovered that only remdesivir significantly decreased viral load at a concentration not toxic to the human cells (therapeutic index of 28.6) [209]. An *in-vivo* study constructed a chimeric mouse-adapted SARS-CoV variant to infect mice with the SARS-CoV-2 RdRp and found that subcutaneous injections of remdesivir resulted in improved lung function and decreased viral load [210].

Human data on the efficacy of these RdRp inhibitors in treating COVID-19 is limited, but there have been some clinical trials as well as studies on previously known diseases that can help judge the potential of some of these drugs. Ribavirin clinical trials against MERS revealed high levels of toxicity indicating that drug may not be the best candidate for COVID-19 treatment [211]. Sofosbuvir with velpatasvir is currently used as an effective hepatitis-C treatment and is well tolerated in patients indicating it may be able to reach effective dosage concentrations to treat COVID-19. Clinical trials of this drug combination are currently underway in Iran [212]. Remdesivir has been used to effectively treat the Ebola and has been used as a COVID-19 treatment for compassionate use in the U.S. and other countries. An observational study analyzing data from 53 patients using remdesivir for compassionate use, found that 68% of patients showed clinical improvement after the first dose and 23% had serious adverse effects [213, 214]. A phase 3 double blinded clinical trial comparing intravenous remdesivir to placebo was completed in Hubei, China. The study consisted of 158 patients in the remdesivir arm and 79 receiving a placebo and concluded that remdesivir was not associated with clinical improvement. Yet, there was a  non-statistically significant trend for quicker recovery times in the intervention group, which may warrant a need for a larger clinical trial [215]. As stated, preliminary analysis of SOLIDARITY trial findings demonstrates no meaningful clinical difference from remdesivir administration compared to the standard of care. Finally, favipiravir is clinically approved for treatment of influenza in Japan and has shown some effectiveness in treating Ebola. This drug has also been used in a randomized control COVID-19 trial in China. The trial was a head to head comparison of favipiravir and arbidol with roughly 120 patients in each arm. The study showed no significant difference between therapies for 7-day clinical recovery rate. Yet, favipiravir did significantly decrease fever and cough symptoms faster and revealed a trend of greater effectiveness on moderately compared to severely ill patients [216, 217]. There are a number of clinical trials currently registered that test these various RdRp inhibitors. One study conducting a phase 4 trial with favipiravir plus HCQ, and multiple trials for remdesivir and sofosbuvir have reached phase 3 but none have published any statistically significant results so far.

### Zinc as a potential RdRp inhibitor

In vitro cell studies have illustrated that zinc directly inhibits the RdRp in SARS-CoV, but a zinc ionophore is needed to move zinc into the cell to be effective [218]. Zinc is known to play an important role in immunomodulation and zinc deficiency is also prevalent amongst high-risk SARS-CoV-2 infectious groups including people of old age, on diuretics, and anti-hypertensive medications [219]. Furthermore, HCQ is a potential drug treatment for COVID-19 as well as a zinc ionophore. Therefore, giving zinc alone and with HCQ has been hypothesized to reduce viral load and attenuate the immune response in SARS-CoV-2 infected patients [220]. Yet, recent studies have illustrated that HCQ is ineffective in reducing infection risk prophylactically or improving outcomes in mild to moderate infections [131, 221]. There are several ongoing clinical trials registered in clinicaltrials.gov aimed to determine whether zinc, along with other agents, is effective in preventing SARS-CoV-2 infection and/or reducing viral load (Additional file 1: Table 1). Trials featuring zinc as a treatment modality in isolation have been sparse and appear to have been discontinued. Unfortunately, as of February 1st, 2021, none of these trials have shown major efficacy with regards to hard endpoints, though many trials are ongoing.

## Conclusions

Human health is under perpetual attack by highly efficient disease vectors that infiltrate our cells, commandeering our own machinery, to wage war on our internal systems. While concentrated efforts have been placed on researching viral mechanisms, there are precious little tools, aside from vaccination, to defend ourselves from the unceasing onslaught of viral attacks. Especially insidious are viral particles that emerge in the human population through zoonotic transfer, such as the recent SARS-CoV [222, 223], MERS [224] and SARS-CoV-2 [3, 214], all of which are betacoronaviruses and have their genetic origin in bat viruses [3, 225–227]. These non-equilibrium viruses have mechanisms specifically adapted to evade the immune system of their ancestor species—mechanisms that our human immune systems have yet evolved to detect [228–231]. As a consequence, non-equilibrium viruses can be deadly, as evidenced in the recent zoonotic transfers mentioned above and the alarmingly high death count from this current and ongoing pandemic.

Alarmingly, over 500 coronaviruses have been identified in bats, and estimates of unidentified coronaviruses are upwards of 5,000, raising the concern that the current pandemic, is a harbinger of possible future zoonotic transfers of highly pathogenic coronaviruses [228, 233–235]. Therefore, while the use of synthetic peptides/proteins, recombinant viral vectors, and prepackaged mRNAs as biological arsenals should produce effective vaccinations for SARS-CoV-2, these immunizations may not be effective against future zoonotic-transfers of coronaviruses. To further complicate matters, SARS-CoV-2, as with all coronaviruses, has an RNA genome and is therefore highly mutable [190, 198]. The heterogeneous nature of RNA genomes complicates the targeting of specific sequences on viral proteins [190]. Thus, development of precision antivirals that target the regions of a coronavirus that are less likely to viably mutate, must be especially emphasized  [160, 236–238].

In this review, we discussed antivirals that inhibit viral entry and viral genome replication, highlighting drugs that target highly conserved domains. A review on the safety and development of SARS-CoV-2 vaccinations was published on June 10th, 2020 by Ma and colleagues, and provides an in-depth analysis of the current vaccine candidates [239]. As stated, our opinion is that amplified efforts should be concentrated into developing drugs aimed at highly conserved regions on viral membrane fusion protein domains. Combined with this, inhibitors targeting the substrate binding region of M^Pro^ and the binding sites on RdRp, can provide a useful arsenal to reduce the spread of the virus. These elements are highly conserved across coronaviruses [162, 198, 240] and have a high barrier to resistance, as mutations in these regions would likely reduce pathogen virulence. Therefore, we hypothesize that using a cocktail of these inhibitors may be a potent, multi-pronged approach to reduce both viral entry and replication of the current SARS-CoV-2, as well as in future, novel zoonotic transfers of betacoronaviruses to humans.

## Supplementary Information

**Additional file 1:** Supplemental Table 1: Table of therapies that target the spike protein and ACE2 binding.

## Data Availability

Not applicable. All relevant data are within the paper.

## Abbreviations

3CLpro: 3C-like proteinase
6-HB: 6 Helix bundle
ACE2: Angiotensin converting enzyme 2
ALI: Acute lung injury
ARB: Angiotensin receptor blocker
ARDS: Acute respiratory distress syndrome
COVID-19: Coronavirus disease 2019
CQ: Chloroquine
FP: Fusion peptide
HCQ: Hydroxychloroquine
HCV: Hepatitis C virus
HDAC2: Histone deacetylase 2
HEK293: Human embryonic kidney cell line
HIV: Human immunodeficiency virus
HR1: Helical heptad repeat 1
IgG: Immunoglobulin G
IgM: Immunoglobulin M
IL-1: Interleukin 1
IL-6: Interleukin 6
IVD: In vitro diagnostic
MERS: Middle eastern respiratory syndrome
MPro: Main protease
nsp: Nonstructural proteins
ORF: Open reading frame
PCR: Polymerase chain reaction
RBD: Receptor binding domain
RdRp: RNA dependent RNA polymerase
RECOVERY: Randomised Evaluation of COVid-19 thERapY
S protein: Spike protein
SARS-CoV-2: Severe acute respiratory syndrome coronavirus 2
TMPRSS2: Transmembrane protease serine 2
TRS: Transcription regulatory sequences
WHO: World health organization
