# Supplementary material for: Precision therapeutic targets for COVID-19
Source: Virol J. 2021 Mar 29;18:66. doi: 10.1186/s12985-021-01526-y (PMC8006140; doi:10.1186/s12985-021-01526-y)
Supplement: Supplementary file 1 — Additional file 1: Supplemental Table 1: Table of therapies that target the spike protein and ACE2 binding. [file 12985_2021_1526_MOESM1_ESM.docx]

**Supplemental Table 1: Table of Therapies that Target the Spike Protein and ACE2 Binding**

| **Treatment** | **Mechanism of Action** | **Name** | **Manufacturer/Sponsor** | **Trial Stage** | **Trial Identifier** |
| --- | --- | --- | --- | --- | --- |
| DNA Vaccine | Viral Spike Protein | INO-4800 | Inovio, CEPI | Phase I/II | NCT04336410 |
|  |  | GX-19 | Genexine, Inc. | Phase I/II | NCT04445389 |
| RNA Vaccine | Viral Spike Protein, Nanoparticle Delivery | mRNA-1273 | NIAID/Moderna | Phase III | NCT04283461 |
|  | Viral Spike Protein, Prime/Boost Dosing | BNT162b2 | BioNTech RNA Pharmaceuticals GmbH, Pfizer | Phase III | NCT04380701 |
|  | Viral Spike Protein | CVnCoV | CureVac AG | Phase I/II | NCT04449276 |
|  | Self-Amplifying S Glycoprotein (+) Sense RNA, Nanoparticle Delivery | LNP-nCoVsaRNA | Imperial College London | Phase I/II | "COVAC1" ISRCTN17072692, EudraCT number: 2020-001646-20 |
| Protein Vaccine | Viral Spike Protein | SARS-CoV-2 rS +/- Matrix-M Adjuvant | NovaVax | Phase III | NCT04368988 |
| Attenuated SARS-CoV-2 Vaccine | Viral Spike Protein | CoronaVac | Sinovac Research and Development Co., Ltd. | Phase III | NCT04352608, NCT04383574 |
| Other Viral Vector Vaccine | Viral Spike Protein | Influenzae Viral Platform, NasoVAX | AltImmune | Phase II | NCT04442230 |
| Bacterial Vector Vaccine | Viral Spike Protein (Plasmid Encoded) | *Bifibidobacterium*  Vector, Plasmid-Encoded Spike Protein Antigen | Symvivo | Phase I | NCT04334980 |
| Recombinant Antigen Vaccines | Trimerization of RNA Virus Spike Proteins +/- Adjuvant | Trimerized Spike Protein (SCB-2019) | Clover Biopharmaceuticals, GlaxoSmithKline | Phase I/II | NCT04405908 |
|  | Recombinant Viral Spike Protein with ADVAX Adjuvant | COVAX-19 | GeneCure Biotechnologies, Vaxine, Medytox | Phase I | NCT04428073, NCT04453852 |

| **Treatment** | **Mechanism of Action** | **Name** | | **Manufacturer/Sponsor** | | **Trial Stage** | | **Trial Identifier** |
| --- | --- | --- | --- | --- | --- | --- | --- | --- |
| Virus-like particle (VLP) Vaccine | Viral Spike Protein Subunit | Viral Subunit VLP | | AFC Biologics, AdaptVac | | Pre-Clinical | | N/A |
| Adenoviral Vector Vaccine | Adv5-Delivered Spike Protein | Adv5 (CTII-nCoV) | | CanSino Biologics Inc, Beijing Institute of Biotechnology, Ministry of Science & Technology China | | Phase III | | "CTCOVID-19" ChiCTR200003178, NCT04313127 |
|  |  |  |  | Jiangsu Provincial CDC, Academy of Military Medical Sciences, China | | Phase III | | ChiCTR200003090, NCT04341389 |
|  | Adv26-Delivered Spike Protein | Adv26 SARS-COV-2 (Ad26COVS1) | | Janssen Vaccine & Prevention, Johnson & Johnson | | Phase III | | NCT04436276 |
|  |  |  |  | Gamaleya Research Institute of Epidemiology and Microbiology, Russian Federation | | Phase III | | NCT04436276 |
|  | Viral Spike Protein | ChAdOx1 nCoV-19/ COV001/AZD1222 | | University of Oxford, AstraZeneca | | Phase III | | PACTR202005681895696, NCT04516746 |
|  |  |  |  | CTRG, UKRI, University of Oxford | | Phase III | | "COV002" 2020-001228-32 |
|  |  |  |  | University of Oxford, UKRI | | Phase III | | "COV001" EudraCT Number: 2020-001072-15 |
| Neutralizing Antibodies | Bind and Sequester Viral Spike Protein | Anti-Spike Protein Antibodies | | Regeneron Pharmaceuticals | | Phase III | | NCT04425629, NCT04426695 |
|  |  |  |  | Hospices Civils de Lyon, Eurobio Scientific | | Phase I | | NCT04354766 |
|  |  |  |  | Tychan Pte Ltd. | | Phase I | | NCT04429529 |
|  |  |  |  | Nantes University Hospital, BPIfrance, Xenothera SAS | | Phase II | | NCT04453384 |
|  |  |  |  | Eli Lilly and Company | | Phase II | | NCT04342897 |
|  |  |  |  | Eli Lilly and Company, AbCellera Biologics Inc. | | Phase II | | "BLAZE-1" NCT04427501 |
| **Treatment** | **Mechanism of Action** | **Name** | **Manufacturer/Sponsor** | | **Trial Stage** | | **Trial Identifier** | |
| Angiotensin II receptor blockers (ARBs) | Blocking ACE2 binding site for viral Spike protein | Valsartan, Losartan, Temlisartan | Klinikum St. Georg | | N/A | | "COVIDAL" DRKS-ID:  DRKS00021732 | |
|  |  |  | Radboudumc, Novartis | | Phase III/IV | | PRAETORIAN-COVID EudraCT Number: 2020-001320-34 | |
|  |  |  | University of Kansas Medical Center | | Phase I | | NCT04335123 | |
|  |  |  | Laboratorio Elea Phoenix S.A. | | Phase IV | | NCT04355936 | |
|  |  |  | The George Institute | | Phase IV | | NCT04394117 | |
|  |  | Lasartan and Spiranolactone | ASSISTANCE PUBLIQUE HÔPITAUX DE MARSEILLE | | Phase III | | "COVIDANCE" EudraCT Number: 2020-001766-11 | |
| Angiotensin-converting enzyme (ACE) Inhibitors | Modulating ACE2 Expression | Captopril | Assistance Publique - Hôpitaux de Paris | | Phase II | | "CAPTOCOVID" EudraCT Number: 2020-001700-42, NCT04355429 | |
| Discontinue  ARB/ACE Inhibitor | Modify ACE2 Expression | Discontinue Medications Targeting Angiotensin Function and Expression | Assistance Publique - Hôpitaux de Paris | | Phase III | | ACORES-2 EudraCT Number: 2020-001381-11 | |
|  |  |  | University Hospital, Gentofte, Copenhagen | | N/A | | NCT04351581 | |
|  |  |  | Medical University Innsbruck | | Phase IV | | NCT04353596 | |
| Angiotensin-converting enzyme 2 (ACE2) Agonist | Blocking ACE2 binding site for viral Spike protein | C21 | Vicore Pharma AB | | Phase II | | NCT04452435 | |
| Recombinant ACE2 | Decoy for ACE2 Activity | Recombinant Human ACE2 (APN01) | APEIRON Biologics AG | | Phase II | | "APN01-01-COVID19" EudraCT Number: 2020-001172-15, NCT04335136 | |

| **Treatment** | **Mechanism of Action** | **Name** | **Manufacturer/Sponsor** | **Trial Stage** | **Trial Identifier** |
| --- | --- | --- | --- | --- | --- |
| Fibrosis Inhibitors | Inhibition of TGF-Beta, Collagen Production, IL-1Beta, and TNF | Pirfenidone | The Third Xiangya hospital of Central South University | Phase 0 | ChiCTR2000031138 |
|  |  |  | The First Affiliated Hospital of Guangzhou Medical University | Phase 0 | ChiCTR2000030892 |
|  | Capain Inhibition | BLD-2660 | Blade Therapeutics, Clinipace Worldwide | Phase II | NCT04334460 |
| Hydroxychloroquine, chloroquine | Prevents Phagolysosome Acidification, Inhibits ACE2/Spike Protein Interaction | Hydroxychloroquine +/- Azithromycin, Zinc | IDIVAL, IDIVAL Instituto de Investigación Sanitaria Valdecilla | Phase III | SANsinCOVID EudraCT Number 2020-001704-42 |
|  |  |  | Military Hospital of Tunis | Phase III | " COVID-Milit" NCT04377646 |
|  |  |  | St. Francis Hospital, New York | Phase IV | NCT04370782 |
|  |  |  | Division de la Lutte Contre le VIH et les IST | Phase III | PACTR202005622389003 |
|  |  |  | ALL INDIA INSTITUTE OF MEDICAL SCIENCES | N/A | CTRI/2020/05/024982 |
|  |  |  | Health Research Council of New Zealand | Phase III | ACTRN12620000457943p |
|  |  |  | Sociedad Española de Farmacia Hospitalaria | Phase III | "QUINAVID" EudraCT Number: 2020-001421-31 |
|  |  | Hydroxychloroquine, Azithromycin, Tocalizumab | INSTITUT DE RECERCA H. SANTA CREU I SANT PAU | Phase III | "IIBSP-COV-2020-23" EudraCT Number: 2020-001442-19 |
|  |  | Chloroquine Phosphate | University College London, Bill and Melinda Gates Foundation | Phase III | EudraCT number: 2020-001402-38 |
|  |  | Chloroquine + Vitamin C | Health Systems Research Institute (HSRI) | Phase IV | TCTR20200404004 |

**Supplemental Table 2: Therapies that target the main protease (M^Pro^)**

| **Treatment Class** | **Treatment Name** | **Manufacturer/Sponsor** | **Trial Stage** | **Trial Identifier** |
| --- | --- | --- | --- | --- |
| HIV Protease Inhibitor | Lopinavir/Ritonavir +/- Interferon Beta | The University of Hong Kong | Phase II | NCT04276688 |
|  |  | Basque Health Service | N/A | EudraCT Number: 2020-001605-23 |
|  |  | University of Queensland, Univeristy of Melbourne | Phase III | "ASCOT" 2020-001635-27 |
|  |  | University of Oxford | Phase II/III | ISRCTN50189673 https://doi.org/10.1186/ISRCTN50189673 |
|  |  | The Fifth Medical Center of the PLA, China | N/A | ChiCTR2000031196 |
|  |  | Istituto Nazionale Per Le Malattie Infettive | Phase II | "ESCAPE" EudraCT Number: 2020-001390-76 |
|  | Darunavir/Cobicistat | Shanghai Public Health Clinical Center | Phase III | NCT04252274 |
|  |  | Hamad Medical Corporation | Phase III | NCT04425382 |
|  | ASC09/ritonavir | Tongji Hospital | Phase III | NCT04261270 |
|  | ASC09/ritonavir, lopinavir/ritonavir | First Affiliated Hospital of Zhejiang University | N/A | NCT04261907 |
| HCV Protease Inhibitor | Danoprevir/Ritonavir | Ascletis Pharmaceuticals Co. | Phase IV | NCT04291729, ChiCTR2000031734,  NCT04345276 |
| Structural M^pro^ Inhibitors | 11a, 11b, N3, α-ketoamides, compound 4, GC376, MAC-5576 etc. | Under development  at multiple institutions | Preclinical | N/A |

**Supplemental Table 3: Therapies that target RNA-dependent RNA polymerase (RdRp)**

| **Treatment Class** | **Mechanism of Action** | **Treatment Name** | **Manufacturer/Sponsor** | **Trial Stage** | **Trial Identifier** |
| --- | --- | --- | --- | --- | --- |
| Nucleoside Analogs | Inhibition of RdRp | Remdesivir | University of Helsinki | Phase III | "WHO-FIN-COVID-19" EudraCT Number: 2020-001784-88 |
|  |  |  | Tehran University | Phase II/III | IRCT20171122037571N2 |
|  |  |  | FIB-HCSC | Phase III | EudraCT Number: 2020-001366-11 |
|  |  |  | Oslo University Hospital | Phase III | "NOR-SOLIDARITY" EudraCT Number: 2020-000982-18 |
|  |  | Sofosbuvir/daclatasvir | Cairo University | Phase II/III | NCT04443725 |
|  |  | Clevudine | Bukwang Pharmaceutical | Phase II | NCT04347915 |
| Influenza Enzyme Inhibitors | Inhibition of RdRp | Favipivir | Fujifilm Pharmaceuticals | Phase II | NCT04358549 |
|  |  |  | Ain Shams University | Phase III | NCT04349241 |
|  |  |  | Shahid Beheshti University of Medical Sciences | Phase IV | NCT04359615 |
|  |  |  | Giuliano Rizzardini | Phase III | NCT04336904 |
|  |  |  | Peking University First Hospital | N/A | NCT04310228 |
|  |  |  | Chromis LLC | Phase II/III | NCT04434248 |
|  |  |  | Stanford University | Phase II | NCT04346628 |
| Vitamin and Mineral Supplementation | Potential RdRp Inhibition, nutrient supplement | Vitamin C + Zinc | The Cleveland Clinic | N/A | NCT04342728 |
|  |  | Zinc | Esfahan University of Medical Sciences | Phase II/III | IRCT20180425039414N2 |
|  |  |  | University of Melbourne | Phase I/II | ACTRN12620000454976 |

**Supplemental Table 4: Therapies that target the SARS-CoV-2 whole viral particle**

| **Treatment Class** | **Treatment Name** | **Manufacturer/Sponsor** | **Trial Stage** | **Trial Identifier** |
| --- | --- | --- | --- | --- |
| Inactivated Whole Virus Vaccines | Heat-Inactivated Plasma Antigens | Immunitor LLC | Phase I/II | NCT04380532 |
|  | CNBG | Sinopharm | Phase III | ChiCTR2000031809 |
| Convalescent Plasma | Infusion of multiple antibodies from Convalescent Plasma | High Educational and Research Institute of Transfusion Medicine | Phase III | N/A |
|  |  | Tongji Medical College, Huazhong University of Science and Technology | Retrospective study | ChiCTR2000033798 |
|  |  | QIMR Berghofer Medical Research Institute | Phase I | ChiCTR2000033056 |
|  |  | King Saud Medical City | N/A | ISRCTN21363594 https://doi.org/10.1186/ISRCTN21363594 |
|  |  | Oroumia University of Medical Sciences | Phase II/III | N/A |
|  |  | Birjand University of Medical Sciences | Phase III | N/A |
|  |  | Eastern theater General Hospital | Prospective Cohort | ChiCTR2000031501 |
|  |  | DRK-Bluspendedienst Baden-Württemberg - Hessen gGmbH, Ministry of Health | Phase III | CAPSID2020-DRK-BSD EudraCT Number: 2020-001310-38 |
|  |  | Medical College of Wisconsin | Phase II | NCT04354831 |
|  |  | Grupo Mexicano para el Estudio de la Medicina Intensiva | Phase II | NCT04405310 |
|  |  | Johns Hopkins University, Cedar-Sinai Medical Center | Phase I | NCT04353206 |
|  |  | The Christ Hospital | Phase I | NCT04355897 |
|  |  | Thomas Jefferson University | Phase II | NCT04389710 |

| **Figure 1** | | |
| --- | --- | --- |
| **Protein** | **PDB ID** | **References** |
| ACE2 | 1R42 | [1] |
| TMPRSS2 | 5CE1 | unpublished |
| Furin | 1P8J | [2] |
| Cathepsin | 6EZX | [3] |
| Spike (Closed) | 6VXX | [4] |
| Spike (Open) | 6VSB | [5] |
| RBD bound to ACE2 | 6MOJ | [6] |
| Transmembrane domain | 5JYN | [7] |
| HR1 | 2FXP | [8] |
| HR2 | 1ZV8 | [9] |
| Fusion Peptide | 2RUM | unpublished |
| **Figure 2** | | |
| **Protein** | **PDB ID** | **References** |
| S2 Fusion Core (1st conformation) | 2BEQ | [10] |
| S2 Fusion Core (2nd conformation) | 1WNC | [11] |
| 6-HB Post-Fusion Core | 6LXT | [12] |
| **Figure 3** | | |
| **Protein** | **PDB ID** | **References** |
| Mpro | 1Q2W | unpublished |
| RdRp | 6M71 | [13] |
| NSP 1 | 2HSX | [14] |
| NSP7 & 8 | 6WIQ | unpublished |
| NSP15 | 6VWW | [15] |
| Envelope | 5X29 | [16] |
| M | 3I6G | [17] |
| Nucleocapsid | 7C22 | unpublished |

**Supplemental Table 5: List of Protein Database (PDB) References**

**References**

[1] Towler P, Staker B, Prasad SG, Menon S, Tang J, Parsons T, et al. ACE2 X-Ray Structures Reveal a Large Hinge-bending Motion Important for Inhibitor Binding and Catalysis. J Biol Chem 2004;279:17996–8007. https://doi.org/10.1074/jbc.M311191200.

[2] Henrich S, Cameron A, Bourenkov GP, Kiefersauer R, Huber R, Lindberg I, et al. The crystal structure of the proprotein processing proteinase furin explains its stringent specificity. Nat Struct Biol 2003;10:520–6. https://doi.org/10.1038/nsb941.

[3] Giroud M, Dietzel U, Anselm L, Banner D, Kuglstatter A, Benz J, et al. Repurposing a Library of Human Cathepsin L Ligands: Identification of Macrocyclic Lactams as Potent Rhodesain and Trypanosoma brucei Inhibitors. J Med Chem 2018;61:3350–69. https://doi.org/10.1021/acs.jmedchem.7b01869.

[4] Walls AC, Park Y-JJ, Tortorici MA, Wall A, McGuire AT, Veesler D. Structure, Function, and Antigenicity of the SARS-CoV-2 Spike Glycoprotein. Cell 2020;181:281-292.e6. https://doi.org/10.1016/j.cell.2020.02.058.

[5] Wrapp D, Wang N, Corbett KS, Goldsmith JA, Hsieh CL, Abiona O, et al. Cryo-EM structure of the 2019-nCoV spike in the prefusion conformation. Science (80- ) 2020;367:1260–3. https://doi.org/10.1126/science.abb2507.

[6] Mohan K, Ueda G, Kim AR, Jude KM, Fallas JA, Guo Y, et al. Topological control of cytokine receptor signaling induces differential effects in hematopoiesis. Science 2019;364:7532. https://doi.org/10.1126/science.aav7532.

[7] Dev J, Park D, Fu Q, Chen J, Ha HJ, Ghantous F, et al. Structural basis for membrane anchoring of HIV-1 envelope spike. Science (80- ) 2016;353:172–5. https://doi.org/10.1126/science.aaf7066.

[8] Hakansson-McReynolds S, Jiang S, Rong L, Caffrey M. Solution structure of the severe acute respiratory syndrome-coronavirus heptad repeat 2 domain in the prefusion state. J Biol Chem 2006;281:11965–71. https://doi.org/10.1074/jbc.M601174200.

[9] Deng Y, Liu J, Zheng Q, Yong W, Lu M. Structures and Polymorphic Interactions of Two Heptad-Repeat Regions of the SARS Virus S2 Protein. Structure 2006;14:889–99. https://doi.org/10.1016/j.str.2006.03.007.

[10] Supekar VM, Bruckmann C, Ingallinella P, Bianchi E, Pessi A, Carfi A. Structure of a proteolytically resistant core from the severe acute respiratory syndrome coronavirus S2 fusion protein. Proc Natl Acad Sci U S A 2004;101:17958–63. https://doi.org/10.1073/pnas.0406128102.

[11] Xu Y, Lou Z, Liu Y, Pang H, Tien P, Gao GF, et al. Crystal structure of severe acute respiratory syndrome coronavirus spike protein fusion core. J Biol Chem 2004;279:49414–9. https://doi.org/10.1074/jbc.M408782200.

[12] Xia S, Liu M, Wang C, Xu W, Lan Q, Feng S, et al. Inhibition of SARS-CoV-2 (previously 2019-nCoV) infection by a highly potent pan-coronavirus fusion inhibitor targeting its spike protein that harbors a high capacity to mediate membrane fusion. Cell Res 2020;30:343–55. https://doi.org/10.1038/s41422-020-0305-x.

[13] Gao Y, Yan L, Huang Y, Liu F, Zhao Y, Cao L, et al. Structure of the RNA-dependent RNA polymerase from COVID-19 virus. Science (80- ) 2020;368:779–82. https://doi.org/10.1126/science.abb7498.

[14] Almeida MS, Johnson MA, Herrmann T, Geralt M, Wüthrich K. Novel β-Barrel Fold in the Nuclear Magnetic Resonance Structure of the Replicase Nonstructural Protein 1 from the Severe Acute Respiratory Syndrome Coronavirus. J Virol 2007;81:3151–61. https://doi.org/10.1128/jvi.01939-06.

[15] Kim Y, Jedrzejczak R, Maltseva NI, Wilamowski M, Endres M, Godzik A, et al. Crystal structure of Nsp15 endoribonuclease NendoU from SARS-CoV-2. Protein Sci 2020;29:1596–605. https://doi.org/10.1002/pro.3873.

[16] Surya W, Li Y, Torres J. Structural model of the SARS coronavirus E channel in LMPG micelles. Biochim Biophys Acta - Biomembr 2018;1860:1309–17. https://doi.org/10.1016/j.bbamem.2018.02.017.

[17] Liu J, Sun Y, Qi J, Chu F, Wu H, Gao F, et al. The membrane protein of severe acute respiratory syndrome coronavirus acts as a dominant immunogen revealed by a clustering region of novel functionally and structurally defined cytotoxic T-lymphocyte epitopes. J Infect Dis 2010;202:1171–80. https://doi.org/10.1086/656315.
